# Economic precarity, loneliness, and suicidal ideation during the COVID-19 pandemic

**DOI:** 10.1371/journal.pone.0275973

**Published:** 2022-11-16

**Authors:** Julia Raifman, Catherine K. Ettman, Lorraine T. Dean, Salma M. Abdalla, Alexandra Skinner, Colleen L. Barry, Sandro Galea

**Affiliations:** 1 Boston University School of Public Health, Boston, MA, United States of America; 2 Johns Hopkins University Bloomberg School of Public Health, Baltimore, MD, United States of America; 3 Brown University School of Public Health, Providence, RI, United States of America; 4 Cornell Jeb E. Brooks School of Public Policy, Ithaca, NY, United States of America; Universidade Federal do Rio Grande do Sul, BRAZIL

## Abstract

The US population faced stressors associated with suicide brought on by the COVID-19 pandemic. Understanding the relationship between stressors and suicidal ideation in the context of the pandemic may inform policies and programs to prevent suicidality and suicide. We compared suicidal ideation between two cross-sectional, nationally representative surveys of adults in the United States: the 2017–2018 National Health and Nutrition Examination Survey (NHANES) and the 2020 COVID-19 and Life Stressors Impact on Mental Health and Well-being (CLIMB) study (conducted March 31 to April 13). We estimated the association between stressors and suicidal ideation in bivariable and multivariable Poisson regression models with robust variance to generate unadjusted and adjusted prevalence ratios (PR and aPR). Suicidal ideation increased from 3.4% in the 2017–2018 NHANES to 16.3% in the 2020 CLIMB survey, and from 5.8% to 26.4% among participants in low-income households. In the multivariable model, difficulty paying rent (aPR: 1.5, 95% CI: 1.2–2.1) and feeling alone (aPR: 1.9, 95% CI: 1.5–2.4) were associated with suicidal ideation but job loss was not (aPR: 0.9, 95% CI: 0.6 to 1.2). Suicidal ideation increased by 12.9 percentage points and was almost 4.8 times higher during the COVID-19 pandemic. Suicidal ideation was more prevalent among people facing difficulty paying rent (31.5%), job loss (24.1%), and loneliness (25.1%), with each stressor associated with suicidal ideation in bivariable models. Difficulty paying rent and loneliness were most associated with suicidal ideation. Policies and programs to support people experiencing economic precarity and loneliness may contribute to suicide prevention.

## Introduction

At the start of the coronavirus 2019 (COVID-19) pandemic, the population of the United States (US) faced several co-occurring stressors. The pandemic led to economic downturn, creating stressors of job loss and financial distress. Black, Hispanic, and Native American people are made especially vulnerable to these economic shocks as a result of centuries of structural racism that shape inequities in wealth [1]. Additionally, physical distancing to prevent the spread of COVID-19 created stressors including social isolation and loneliness.

Economic precarity [2] and social isolation [3] are associated with mental distress and suicide. Other studies have documented increases in mental distress and suicidality [4–8] during the pandemic, but few have examined economic hardship. While there were not increases in suicide deaths in the US or many other countries in 2020 [9–11], suicide remains a leading cause of premature death in the US [12] and may continue to evolve [13]. Suicidality is an indicator of major mental distress and a risk factor for suicide [14]. Understanding the populations most at risk of suicidal ideation and the association between COVID-19 stressors and suicidal ideation can inform policies and programs to reduce suicidality and prevent suicide. This study aimed to evaluate the relationship between stressors and suicidal ideation during the start of the COVID-19 pandemic.

## Methods

### Sample

We used data from a nationally representative sample of US adults aged 18 or older collected through the AmeriSpeak standing panel. Panelists were invited to participate in the COVID-19 and Life Stressors Impact on Mental Health and Well-being (CLIMB) study from March 31, 2020 through April 13, 2020 and were paid a cash equivalent of $3 for completing the survey (64.3% completion rate). We created and applied post-stratification weights to align the study sample with the U.S. adult population according to the U.S. Current Population Survey [15]. Previously published work further describes details on the AmeriSpeak sampling frame and the CLIMB study [5,16]. As a pre-pandemic comparison, we used data from the 2017–2018 National Health and Nutrition Examination Survey (NHANES), a nationally representative sample of noninstitutionalized civilian US adults aged 18 years or older collected by the US government. The CLIMB and NHANES samples are comparable in that both are nationally representative groups of US adults residing in all 50 states and the District of Columbia. The AmeriSpeak sampling frame covers approximately 97% of all US households, and NHANES sampling units similarly cover all US counties. We excluded participants who did not respond to questions about suicidal ideation in NHANES and participants who did not respond to any analysis variables in CLIMB data.

### Exposures

We evaluated three COVID-19 stressors reflecting economic precarity and loneliness, each measured as binary variables reported in response to a question, “Have any of the following affected your life as a result of the coronavirus or COVID-19 outbreak?” Our exposure variables were based on whether participants checked boxes for “losing a job,” “having difficulty paying rent,” and “feeling alone”.

### Outcome

Both surveys assessed suicidal ideation based on Patient Health Questionnaire-9 (PHQ-9) item 9, which asks participants whether they have had “Thoughts that you would be better off dead or of hurting yourself in some way” over the past two weeks. Response options are “Not at all,” “several days,” “more than half the days,” or “nearly every day”. We created a binary variable for reporting these feelings with any frequency. Prior research indicates responses to this question were correlated with future suicide attempts and deaths [14,17,18].

### Analysis

First, we described the demographic characteristics of participants in the 2020 CLIMB data and in the 2017–2018 NHANES data. Second, we estimated the prevalence of suicidal ideation by demographic characteristics and calculated the share with suicidal ideation within subgroups in 2020 relative to 2017–2018. Third, we used CLIMB data to estimate unadjusted and adjusted prevalence ratios (PR and aPR) of the association between COVID-19 related stressors and suicidal ideation using a Poisson regression model with robust variance to approximate a log-binomial regression model, with α = 0.05 [19]. In the multivariable model, to reduce bias, we adjusted for age group, education level, sex, race and ethnicity, household income, savings, marital status, COVID-19 illness, and COVID-19 bereavement. We ran a multivariate sensitivity analysis without the stressor variable for “having difficulty paying rent” in the same regression model as the “losing a job” exposure because difficulty paying rent may lie on the causal pathway in the association between job loss and suicidal ideation.

## Results

A total of 1,415 (96.3%) of 1,470 CLIMB participants responded to all questions relevant to the analysis and 5,085 (86.8%) of 5,856 NHANES participants responded to suicidal ideation questions and were included in the samples (Table 1). The NHANES sample was younger, less likely to be married, more likely to have high school or less education, and less likely to be low-income relative to the CLIMB sample.

**Table 1:** Participant demographic characteristics.

	Sample characteristics				
	NHANES, 2017–2018		CLIMB, 2020		p-value
	n	%	n	%	
Total	5,085	100	1,415	100	N/A
**Difficulty paying rent**					
No	N/A	N/A	1,199	84.7	N/A
Yes	N/A	N/A	216	15.3	N/A
**Lost job**					
No	N/A	N/A	1,253	88.5	N/A
Yes	N/A	N/A	162	11.5	N/A
**Feeling alone**					
No	N/A	N/A	952	67.3	N/A
Yes	N/A	N/A	463	32.7	N/A
Mean age (avg, std)	49.6	18.5	46.0	16.5	N/A
Age group					
18–29	965	21.3	238	16.8	<0.001
30–44	1,106	24.2	493	34.8	<0.001
45–59	1,183	26.5	337	23.8	0,040
60+	1,831	28.1	347	24.5	<0.001
Sex					
Male	2,489	48.7	708	50.0	0.387
Female	2,596	51.3	707	50.0	0.387
Race/ethnicity					
Non-Hispanic White	1,801	63.0	922	65.2	0.129
Non-Hispanic Black	1,178	11.1	137	9.7	0.133
Non-Hispanic another race or multiracial	947	10.1	105	7.4	0.002
Hispanic	1,159	15.8	251	17.7	0.086
Education level					
High school graduate or less	2,262	39.4	327	23.1	<0.001
Some college	1,640	30.4	631	44.5	<0.001
College grad or more	1,177	30.2	457	32.3	0.130
Not reported	6	<0.1	0	N/A	N/A
Marital status					
Unmarried or separated	2,424	45.1	716	50.6	<0.001
Married	2,412	51.5	699	49.4	0.162
Not reported	249	3.4	0	N/A	<0.001
Household income					
$0-$19,999	875	11.5	220	15.6	<0.001
$20,000-$39,999^a^	1,322	21.4	321	22.7	0.294
$40,000-$74,999^a^	891	17.7	410	29.0	<0.001
≥$75,000	1,356	38.7	464	32.8	<0.001
Not reported	641	10.7	0	N/A	<0.001
Savings					
<$5,000	N/A	N/A	720	50.9	N/A
≥$5,000	N/A	N/A	695	49.1	N/A
COVID-19 illness					
No	N/A	N/A	1,403	99.1	N/A
Yes	N/A	N/A	12	0.9	N/A
Death of someone close due to COVID-19					,
No	N/A	N/A	1,390	98.2	N/A
Yes	N/A	N/A	25	1.8	N/A

Notes: Percents are weighted. Data on suicidal ideation among those with COVID-19 illness and bereavement are based on small sample sizes.

^a^ Income categories for NHANES participants were $20,000 to $44,999 and $45,000 to $74,999 based on different cut points for income questions.

**Fig 1:**
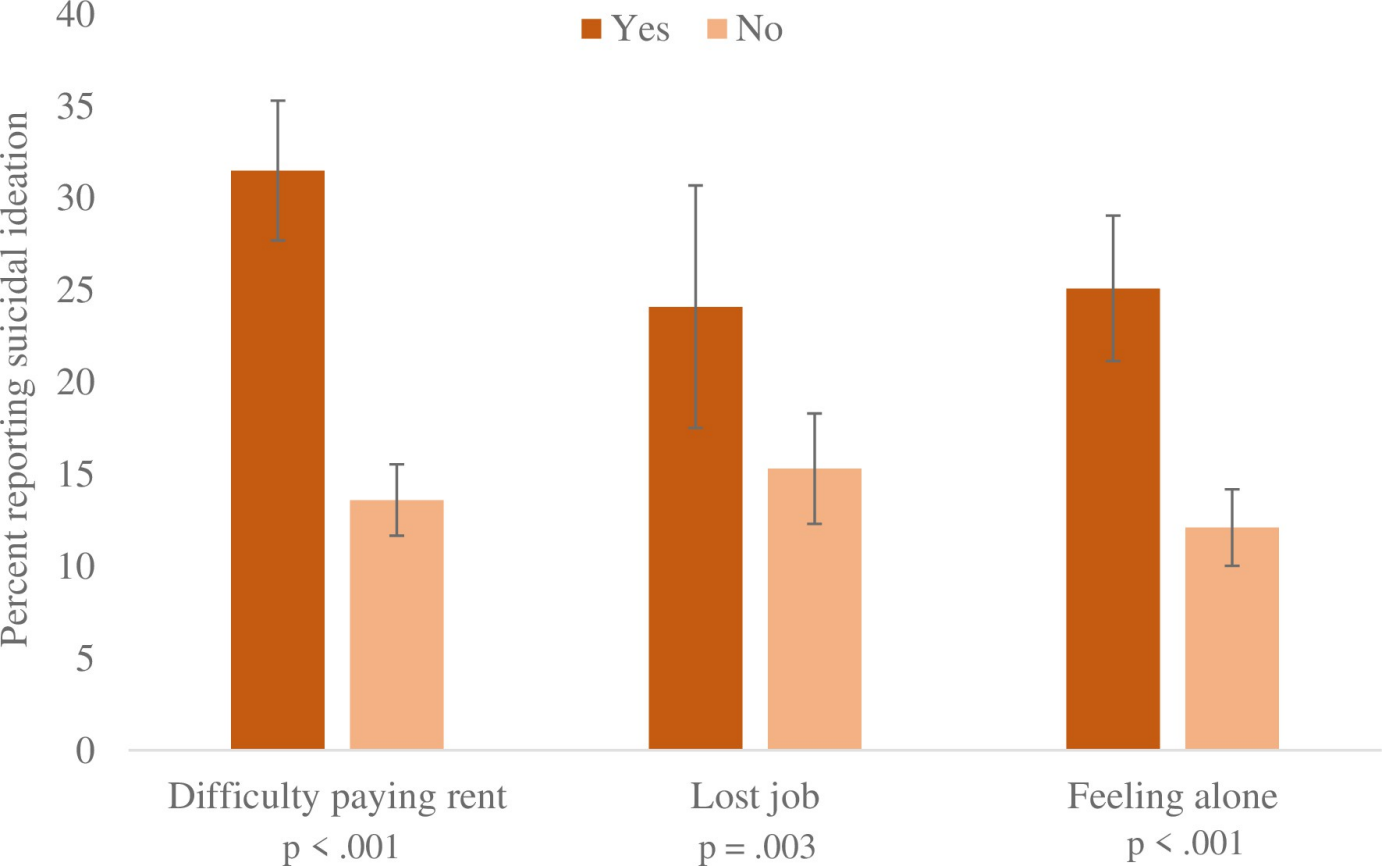
COVID-19 stressors and suicidal ideation. The figure depicts the percentages with 95% confidence intervals of participants who reported suicidal ideation among those who did or did not report COVID-19-related stressors, including difficulty paying rent, losing a job, and feeling alone. Data used to create the figure comes from the COVID-19 and Life Stressors Impact on Mental Health and Well-being (CLIMB) study conducted from March 31, 2020 through April 13, 2020.

The prevalence of suicidal ideation was 3.4% in the 2017–2018 NHANES sample and 16.3% in the 2020 CLIMB survey (Table 2). The greatest absolute increases in suicidal ideation and 2020 prevalence of suicidal ideation were among participants earning less than $20,000 (5.8% to 26.4%), participants aged 18 to 29 (4.1% to 23.5%), and participants who were Hispanic (3.7% to 23.1%). In 2020, suicidal ideation was high among those who faced difficulty paying rent (31.5%, Fig 1) and job loss (24.1%), as well as loneliness (25.1%).

**Table 2:** COVID-19 stressors, demographic characteristics, and suicidality.

	Suicidal ideation				Absolute difference 2020 - (2017–2018)	Ratio, 2020: 2017–2018	Z score p-value
	NHANES, 2017–2018, Suicidal ideation		CLIMB, 2020				
	n	%	n	%	Percentage points		
Total	192	3.4	231	16.3	12.9	4.8	<0.001
**Difficulty paying rent**							
No	N/A	N/A	163	13.6	N/A	N/A	N/A
Yes	N/A	N/A	68	31.5	N/A	N/A	N/A
**Lost job**							
No	N/A	N/A	192	15.3	N/A	N/A	N/A
Yes	N/A	N/A	39	24.1	N/A	N/A	N/A
**Feeling alone**							
No	N/A	N/A	115	12.1	N/A	N/A	N/A
Yes	N/A	N/A	116	25.1	N/A	N/A	N/A
Age group							
18–29	38	4.1	56	23.5	19.1	5.7	<0.001
30–44	38	3.1	92	18.7	15.4	6.0	<0.001
45–59	48	3.0	47	14.0	11	4.7	<0.001
60+	68	3.5	36	10.4	6.8	2.9	<0.001
Sex							
Male	102	4.0	109	15.4	11.5	3.9	<0.001
Female	90	2.9	122	17.3	13.9	5.8	<0.001
Race/ethnicity							
Non-Hispanic White	71	3.3	124	13.5	10.2	4.1	<0.001
Non-Hispanic Black	33	3.1	22	16.1	12.1	4.9	<0.001
Non-Hispanic another race or multiracial	34	4.0	27	25.7	20.1	6.0	<0.001
Hispanic	54	3.7	58	23.1	19.1	6.2	<0.001
Education level							
High school graduate or less	112	4.2	68	20.8	16.1	4.8	<0.001
Some college	60	4.3	101	16	11.8	3.7	<0.001
College grad or more	20	1.4	62	13.6	11.7	9.4	<0.001
Not reported	N/A	N/A	N/A	N/A	N/A	N/A	
Marital status							
Unmarried or separated	127	4.6	152	21.2	16.4	4.6	<0.001
Married	54	2.2	79	11.3	8.9	5.0	<0.001
Not reported	11	5.2	N/A	N/A	N/A	N/A	N/A
Household income							
$0-$19,999	50	5.8	58	26.4	19.8	4.4	<0.001
$20,000-$39,999^a^	63	5.0	56	17.5	12.1	3.4	<0.001
$40,000-$74,999^a^	32	3.3	59	14.4	11.1	4.4	<0.001
≥$75,000	25	2.1	58	12.5	10.5	6.0	<0.001
Not reported	22	2.7	N/A	N/A	N/A	N/A	N/A
Savings							
<$5,000	N/A	N/A	155	21.5	N/A	N/A	N/A
≥$5,000	N/A	N/A	76	10.9	N/A	N/A	N/A
COVID-19 illness							
No	N/A	N/A	223	15.9	N/A	N/A	N/A
Yes	N/A	N/A	8	66.7	N/A	N/A	N/A
Death of someone close due to COVID-19							
No	N/A	N/A	222	16	N/A	N/A	N/A
Yes	N/A	N/A	9	36	N/A	N/A	N/A

Notes: Percents are weighted. Data on suicidal ideation among those with COVID-19 illness and bereavement are based on small sample sizes. P-values depict Z-score differences for two sample tests of proportions.

^a^ Income categories for NHANES participants were $20,000 to $44,999 and $45,000 to $74,999 based on different cut points for income questions.

Each of the stressors we evaluated were associated with suicidal ideation in the bivariable model (Table 3; Difficulty paying rent PR: 2.3, 95% CI: 1.8 to 3.1; feeling alone PR: 2.1, 95% CI: 1.6 to 2.6; job loss PR: 1.6, 95% CI: 1.1 to 2.2). In the multivariable model, difficulty paying rent was associated with suicidal ideation (aPR: 1.5, 95% CI: 1.2 to 2.1), while losing a job was not (aPR: 0.9, 95% CI: 0.6 to 1.2). The sensitivity analysis with job loss as the exposure without difficulty paying rent in the model did not change the effect estimate. Feeling alone was also associated with suicidal ideation (aPR: 1.9, 95% CI: 1.5 to 2.4). Although the sample size for persons with COVID-19 illness (n = 12) or bereavement (n = 25) is small, the results (66.7% and 36.0%, respectively) are suggestive that COVID-19 illness or bereavement may be associated with increased suicidal ideation.

**Table 3:** COVID-19 related stressors and suicidal ideation over the past two weeks (N = 1415).

Variables	PR	95% CI	p-value	aPR	95% CI	p-value
**COVID-19 related stressors**						
Lost job	1.6	1.1–2.2	0.004	0.9	0.6–1.2	0.525
Difficulty paying rent	2.3	1.8–3.1	<0.001	1.5	1.2–2.1	0.003
Feeling alone	2.1	1.6–2.6	<0.001	1.9	1.5–2.4	<0.001
**Age group**						
18 to 29	2.3	1.5–3.3	<0.001	1.3	0.9–2.0	0.217
30 to 44	1.8	1.3–2.6	0.001	1.3	0.9–1.9	0.147
45 to 59	1.3	0.9–2.0	0.155	1.1	0.7–1.7	0.627
≥60	Reference group			Reference group		
**Sex**						
Male	Reference group			Reference group		
Female	1.1	0.9–1.4	0.345	0.9	0.7–1.1	0.414
**Race/ethnicity**						
Non-Hispanic White	Reference group			Reference group		
Non-Hispanic Black	1.2	0.8–1.8	0.404	0.8	0.5–1.2	0.288
Non-Hispanic and another race or multiracial	1.9	1.3–2.8	0.001	1.8	1.2–2.6	0.002
Hispanic	1.7	1.3–2.3	<0.001	1.4	1.0–1.8	0.032
**Income group**						
$0-$19,999	2.1	1.5–2.9	<0.001	1.2	0.8–1.8	0.317
$20,000-$39,999	1.4	1.0–2.0	0.054	1.0	0.7–1.4	0.859
$40,000-$74,999	1.2	0.8–1.6	0.414	1.0	0.7–1.4	0.872
≥$75,000	Reference group			Reference group		
**Education level**						
High school graduate or less	1.5	1.1–2.1	0.008	1.2	0.9–1.7	0.271
Some college	1.2	0.9–1.6	0.268	1.0	0.7–1.4	0.965
College graduate or above	Reference group			Reference group		
**Marital status**						
Unmarried or separated	1.9	1.5–2.4	<0.001	1.3	1.0–1.8	0.036
Married	Reference group			Reference group		
**Savings**						
<$5,000	2.0	1.5–2.5	<0.001	1.4	1.1–1.9	0.021
≥$5,000	Reference group			Reference group		
**COVID-19 illness**						
No	Reference group			Reference group		
Yes	4.2	2.8–6.4		3.5	1.9–6.4	<0.001
**Death of someone close due to COVID-19**						
No	Reference group			Reference group		
Yes	2.3	1.3–3.9		1.7	0.9–3.0	0.109

Notes: Estimates are prevalence ratios (PR) and adjusted prevalence ratios (aPR) based on Poisson regression analyses with robust variance. The PR is based on a bivariable analyses of each variable and suicidal ideation. The aPR is adjusted for all variables listed in the table. There were small samples of participants with COVID-19 illness (n = 12) and bereavement (n = 25).

## Discussion

We found that suicidal ideation was 4.8 times higher during the COVID-19 pandemic; 16.1% of people reported suicidal ideation in 2020, relative to 3.4% in 2017–2018. In keeping with prior studies, we found that people living in low-income households are particularly at risk of mental distress during the COVID-19 pandemic [5,6,16].

Reporting difficulty paying rent was associated with suicidal ideation. Prior research indicates that financial distress, such as that which became widespread during the COVID-19 pandemic, is associated with suicide^2^ and that eviction in particular is associated with suicide [20]. Policies such as the Centers for Disease Control and Prevention’s federal eviction moratorium, state eviction moratoriums, and federal and state unemployment insurance policies [21] and federal stimulus payments may help prevent suicide. While job loss was not associated with suicidal ideation during the CLIMB study period of late March and early April in the adjusted analysis, it is important to study job loss and mental health over a longer term period as high unemployment was prolonged for several months, especially for people in low-income households and who are Black and Hispanic [22]. It is also possible that the suicidality impacts of job loss differed by wealth and whether people who lost work faced imminent economic hardship, and further studies among subgroups are needed.

People who reported feeling alone were 1.9 times as likely to report suicidal ideation, highlighting the need for programs and policies to provide social support, such as through social connections in environments with lower COVID-19 risk (e.g. outdoors) or via computer or phone. There is a need for further research on loneliness among subgroups such as older populations.

This study was conducted early in the course of COVID-19 spread across the US; we did not have a large enough sample of persons experiencing COVID-19 illness or bereavement to study the association between COVID-19 illness or bereavement with suicidal ideation. The results suggest a potential association between these exposures and suicidal ideation that warrants further study.

Finally, prior studies indicate that means restriction [23], particularly of firearms [24], is associated with reductions in suicide. Suicide by firearm is the predominant means of suicide death in the United States. As such, and in light of previous evidence that links suicidality to suicide death, policies or programs to reduce household firearm ownership could play an important role in suicide prevention in the COVID-19 context of elevated stressors.

Limitations include that the characteristics of participants in CLIMB and NHANES differed, although both were nationally representative surveys and should be generalizable to the broader population. The NHANES sample was younger and less likely to be low-income. We were further limited by the questions asked in the surveys as indicators of economic precarity and loneliness and the possibility that participants interpreted these questions in different ways. These questions were not included in the NHANES survey, so it was not possible to compare these exposures before and after the pandemic. Furthermore, the CLIMB study was conducted early in the pandemic, and the relationship between stressors and suicidal ideation may have changed as the pandemic and associated stressors continue to affect the US population. There is a need for further research on exposures and suicidality over time and among subgroups, especially those affected by structural racism and inequities.

## Conclusion

Suicidal ideation increased substantially during the COVID-19 pandemic. Those facing difficulty paying rent and feeling alone may be at particular risk of suicide. Policies and programs to support people experiencing economic precarity and difficulty paying rent may contribute to suicide prevention, as may programs to support individuals facing prolonged social isolation.

## Supporting information

S1 Checklist STROBE statement—checklist of items that should be included in reports of *cross-sectional studies*. (DOCX)
